# EGFR^vIII^: An Oncogene with Ambiguous Role

**DOI:** 10.1155/2019/1092587

**Published:** 2019-12-16

**Authors:** Adrianna Rutkowska, Ewelina Stoczyńska-Fidelus, Karolina Janik, Aneta Włodarczyk, Piotr Rieske

**Affiliations:** ^1^Department of Tumor Biology, Medical University of Lodz, Zeligowskiego 7/9, 90-752 Lodz, Poland; ^2^Department of Research and Development, Celther Polska Ltd., Milionowa 23, 93-193 Lodz, Poland; ^3^Department of Research and Development, Personather Ltd., Milionowa 23, 93-193 Lodz, Poland

## Abstract

Epidermal growth factor receptor variant III (EGFR^vIII^) seems to constitute the perfect therapeutic target for glioblastoma (GB), as it is specifically present on up to 28–30% of GB cells. In case of other tumor types, expression and possible role of this oncogene still remain controversial. In spite of EGFR^vIII^ mechanism of action being crucial for the design of small active anticancer molecules and immunotherapies, i.e., CAR-T technology, it is yet to be precisely defined. EGFR^vIII^ is known to be resistant to degradation, but it is still unclear whether it heterodimerizes with EGF-activated wild-type EGFR (EGFR^WT^) or homodimerizes (including covalent homodimerization). Constitutive kinase activity of this mutated receptor is relatively low, and some researchers even claim that a nuclear, but not a membrane function, is crucial for its activity. Based on the analyses of recurrent tumors that are often lacking EGFR^vIII^ expression despite its initial presence in corresponding primary foci, this oncogene is suggested to play a marginal role during later stages of carcinogenesis, while even in primary tumors EGFR^vIII^ expression is detected only in a small percentage of tumor cells, undermining the rationality of EGFR^vIII^-targeting therapies. On the other hand, EGFR^vIII^-positive cells are resistant to apoptosis, more invasive, and characterized with enhanced proliferation rate. Moreover, expression of this oncogenic receptor was also postulated to be a marker of cancer stem cells. Opinions regarding the role that EGFR^vIII^ plays in tumorigenesis and for tumor aggressiveness are clearly contradictory and, therefore, it is crucial not only to determine its mechanism of action, but also to unambiguously define its role at early and advanced cancer stages.

## 1. EGFR: Parental Gene of EGFR^vIII^

Epidermal growth factor receptor (EGFR/ErbB1/HER1) is a member of a tyrosine kinase receptor family, also including ErbB2/HER2/Neu, ErbB3/HER3, and ErbB4/HER4 [1]. All these receptors are transmembrane glycoproteins with a molecular mass ranging from 170 to 185 kDa [2]. Activation of ErbB receptor may be triggered by one of 13 ligands, such as epidermal growth factor (EGF), transforming growth factor-*α* (TGF-*α*), amphiregulin, betacellulin, epiregulin, neuregulin 1–6, heparin-binding EGF-like growth factor (HB-EGF), or epigen, with the first five being EGFR-specific [3]. It is not clear how EGFR is activated and triggers a cascade of downstream signaling in cells. Generally, its activation involves ligand binding and subsequent receptor dimerization; however, it was also indicated that receptor may dimerize regardless of ligand presence [4, 5]. Intriguingly, dimers formed in such a ligand-independent manner remain inactive till the ligand is finally bound [4]. Activation of EGF receptor may induce signal in Ras/Raf/MAPK, PI3K/AKT, JAK/STAT, or PLC/PKC pathways [6, 7], having an impact on a variety of cellular processes, including proliferation, metabolism, apoptosis, cell survival, or differentiation [8, 9]. Termination of signaling cascade occurs after receptor internalization, mostly in clathrin-dependent endocytosis, leading to its trafficking into early endosomes. Further, receptor may be either transported back to the cell membrane or degraded in late endosomes and lysosomes [10].

**Figure 1:**
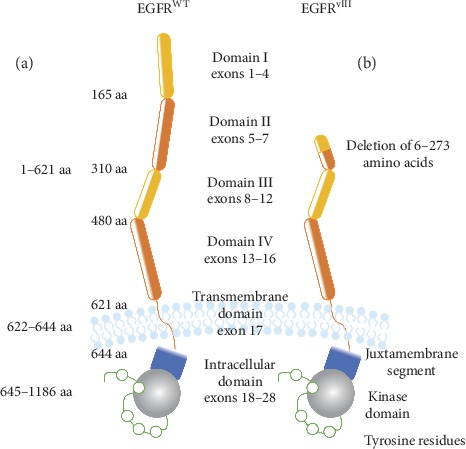
Schematic structure of EGFR^WT^ (a) and EGFR^vIII^ (b). Both receptors are composed of extracellular (I–IV), transmembrane, and intracellular domains, and the major difference is deletion of exons 2–7 encoding extracellular domains I and II in mutated receptor. As a result of deletion, EGFR^vIII^ is unable to bind known ligands and shows enhanced stability in cell membrane.

Gene encoding EGFR is located on a short arm of chromosome 7 (p11.2) and consists of 28 exons [11]. Mature EGFR protein (1186 amino acids) is formed from a precursor one (1210 amino acids) following the removal of the *N*-terminal part [12]. From the *N* to the *C*-terminal end, EGFR is composed of extracellular domain involved in ligand binding and receptor dimerization (exons 1–16), hydrophobic transmembrane domain (exon 17), and intracellular domain with tyrosine kinase activity that is flanked by the linker region and the *C*-terminal part of the receptor (exons 18–28) (Figure 1(a)) [13]. Twelve out of 20 tyrosine residues of intracellular domain were demonstrated to undergo phosphorylation and these bind membrane-bound or cytoplasmic effector proteins that are recruited following receptor activation [14, 15].

## 2. EGFR^vIII^ Alteration in Cancer

Overexpression of EGF receptor was detected in many tumor types and demonstrated to be associated with cancer cell resistance to chemo-, radio-, and/or hormone therapy. This receptor is often mutated in certain tumors, especially in extracellular and tyrosine kinase domains [16], resulting in elevated or prolonged EGFR signaling [17, 18]. Such abnormal signaling is associated not only with enhanced proliferation and apoptosis inhibition in tumor cells, but also with metastasis and angiogenesis [19, 20]. In case of glioblastoma (GB), EGFR amplification is in the majority of cases accompanied by gene rearrangements. Such alterations involve deletion of particular exons or exon parts and are designated as EGFR^vI^ (deletion of *N*-terminal part), EGFR^vII^ (deletion of exons 14 and 15), EGFR^vIII^ (deletion of exons 2–7), EGFR^vIV^ (deletion of exons 25–27), and EGFR^vV^ (deletion of exons 25–28) [21–24]. One of the most commonly detected variants in GB cells is EGFR^vIII^ [25–29] (Figure 1(b)).

Despite being mentioned in several articles [30], alternative splicing does not constitute key mechanisms for EGFR^vIII^ expression in glioblastoma and other tumor types. There are only single reports indicating that this phenomenon may be involved in EGFR^vIII^ generation in head and neck squamous cell carcinoma (HNSCC), but it is still not considered the major mechanism. Gene encoding EGFR is amplified in approximately 50% of GB patients, and in 50–60% of cases, amplification is accompanied by EGFR^vIII^ expression that is tumor cell-specific, making this oncogenic protein a perfect therapeutic target [22, 31, 32]. Expression of mutated receptor is also detected in few percent of prostate, breast, or colon cancer cases, but only in trace cell populations [33–36]. Nevertheless, EGFR^vIII^ expression in tumor types other than glioblastoma remains controversial and needs to be unequivocally assessed, as many contradictory data have been published so far [27, 37–41]. Such inconsistencies are particularly associated with technical limitations of applied methodological approaches. Data collected from several research centers indicate that results of EGFR^vIII^-related analyses tend to be even completely inconsistent. As an example, a research conducted by Moscatello et al. (1995) demonstrated EGFR^vIII^ expression in 73% of ovarian cancer samples (Western blot analysis) and was completely contradictory to independent analysis, utilizing other methods, that indicated lack of this oncogene expression at both mRNA and protein levels in analyzed tumor samples, as well as cell lines [34, 42–44]. Similar inconsistencies were detected in case of colon or bladder cancer [43, 45–47] and most interestingly, in breast cancer, in which case EGFR^vIII^ expression is in some reports estimated to be 20–78%, while in others not to exceed 0–4% [34, 36, 43, 48–50]. Nevertheless, various agents acting on EGFR^WT^ or EGFR^vIII^ are extensively studied in different types of cancers (summarized in Table 1).

From a therapeutic point of view, glioblastoma seems to be the most important tumor type in terms of EGFR^vIII^ because of the relatively high expression and frequency of occurrence of this oncogene and, most importantly, continuous lack of effective therapy for GB patients. Due to the deletion of 801 bp encoding *N*-terminal, domains I and II are lost and the mutated receptor becomes unable to bind ligands [8, 25]. Mechanisms leading to the formation of nucleotide sequence encoding EGFR^vIII^ have not been completely elucidated yet; however, it seems plausible that deletion of receptor part is the result of recombination between Alu sequences flanking junctions in introns 1 and 7 of EGFR-encoding gene [117]. As EGFR^vIII^ usually acts as an amplified gene, it may be suggested that increase in the number of gene copies will translate into increased mRNA levels of this oncogenic variant, but no such obvious dependence has been found, even in relation to EGFR^WT^ levels [118, 119]. It may be associated with the fact that the main role of gene amplification in this case is not to provide additional gene copies that will increase mRNA levels of amplified gene. With the current focus on the field of extracellular vesicles (EVs), the fact that extrachromosomal amplicons may be transported between cells is gaining importance [120]. Therefore, in such a context, the role of amplicons is not to increase oncogene mRNA levels, but rather to enable more flexible regulation of gene expression as well as transfer of mutated gene to cells initially lacking such alteration. Moreover, detection of EGFR^vIII^ in extrachromosomal amplicons derived from cerebrospinal fluid may constitute a highly specific and less invasive approach, to molecularly diagnose GB patients and make them candidates for currently developed anti-EGFR^vIII^-targeted therapies [118].

## 3. EGFR^vIII^ Mechanism of Action

Compared to EGFR^WT^ (normal EGFR protein), EGFR^vIII^ signaling is considered to be elevated, due to its ability to dimerize in a ligand-independent manner. However, as it is not clear whether EGF is indeed crucial for EGFR^WT^ dimerization or required only for a dimer to switch from inactive to active state, the role of EGFR^vIII^ dimerization is becoming less evident [3, 4]. Loss of large extracellular receptor fragment makes it difficult to determine whether EGFR^vIII^ dimerizes in tethered or untethered conformation or if such conformation resembles active or rather inactive EGFR^WT^ [16]. Importantly, it has to be emphasized that EGFR^vIII^ exhibits constitutive activity [121, 122]. Our team demonstrated that mutant phosphorylation is elevated when compared to nonstimulated EGFR^WT^, while data obtained by other research teams indicate that constitutive EGFR^vIII^ signaling corresponds to low level of signal intensity induced by ligand-activated EGFR^WT^ [17, 123–125]. Such data indicate that EGFR^vIII^ dimer conformation resembles inactive dimers of EGFR^WT^, thus suggesting that impact of this oncogenic receptor on cell biology is not a result of some specific, dimerization-related kinase activity, but rather a consequence of unique membrane stability [121, 122]. Therefore, constitutive activity of EGFR^vIII^ is not particularly high, but when combined with high membrane stability, it may enable triggering of some significant biological effects by this oncogenic receptor [17, 123–125].

Despite the fact that EGFR^vIII^ stability seems to be more important than its kinase activity, the latter feature is still required to fully exhibit the oncogenic potential of this receptor. Such potential is dependent on signal transduction induced by phosphorylated tyrosines (at least in a model explaining EGFR^vIII^ oncogenicity as a membrane receptor), especially as EGFR^vIII^ was demonstrated to undergo constant phosphorylation and dephosphorylation cycles [123]. Additionally, our data indicate that EGFR^vIII^ signaling may not be associated with slightly elevated kinase activity, but rather its minimally lower sensitivity to phosphatase activity, when compared to wild-type receptor [123]. Moreover, we indicated that enhanced phosphorylation of tyrosine 1045 did not result in EGFR^vIII^ degradation [123], suggesting that the previous model of impaired EGFR^vIII^ degradation requires an update [121]. Nevertheless, without membrane stability, EGFR^vIII^ signaling will not be strong enough to induce a biological effect. So far, the reasons behind the unique EGFR^vIII^ stability have not been fully elucidated. Initially, it was suggested that it is a result of different phosphorylation of tyrosine responsible for interaction with Cbl protein in mutated and wild-type receptors [121]. Additionally, the involvement of FHL2 in EGFR^WT^ and EGFR^vIII^ stabilization was suggested [126]. Stabilization and activity of EGFR^vIII^ are mostly determined by the quaternary structure of the receptor. Long noncoding RNA (lncRNA) EGFR-AS1, an antisense transcript of EGFR, was suggested to be involved in EGFR folding [127], but it is still unknown whether this structure may have a different impact on mutated than wild-type receptor. Nevertheless, gene encoding this lncRNA is located on the same amplicon as EGFR and thus also undergoes amplification [127]. Actually, it is still unknown why EGFR^vIII^ is much more stable than EGFR^WT^; however, such feature of this oncogenic receptor may be considered crucial, as the ability to trigger EGFR^vIII^ degradation, considering its low kinase activity, may deprive this variant of oncogenic properties. Intriguingly, the impact of the dimerization process alone was suggested to be associated with increased EGFR^vIII^ stability, especially since the involvement of the so-called “crypto” domains was described to have an impact on EGFR^WT^ stabilization [128–132].

**Figure 2:**
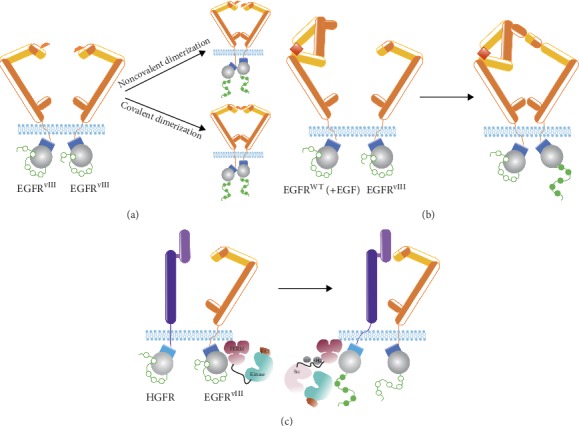
Currently proposed models of EGFR^vIII^ dimerization. (a) Covalently or noncovalently linked EGFR^vIII^ homodimers. In both cases, phosphorylation of tyrosine residues of both monomers can be observed. (b) Heterodimerization of EGFR^vIII^ with ligand-activated (e.g., EGF-activated) EGFR^WT^. Only EGFR^vIII^ phosphorylation is observed in such a case. (c) EGFR^vIII^ dimers with monomers of other inactive receptors. Example of FAK-mediated EGFR^vIII^ dimerization with HGFR, resulting in phosphorylation of HGFR tyrosine residues.

According to the majority of analyses, EGFR^vIII^ is able to form both hetero- and homodimers [123, 133–140], and in the latter case, covalent and noncovalent dimers can be observed [17, 123]. Our analyses indicate that the most of EGFR^vIII^ monomers are a part of covalent homodimers with covalent bonds formed with the involvement of free cysteine in position 16 of amino acid chain (Figure 2(a)) [123]. Nevertheless, some authors undermine the constitutive activity of the mutant, suggesting that EGFR^vIII^ activity is mostly due to EGF-activated EGFR^WT^ forming a heterodimer with EGFR^vIII^ (Figure 2(b)) [138]. Additionally, it was suggested that only EGF-bound EGFR^WT^ is able to phosphorylate EGFR^vIII^ in a heterodimer, but not vice versa [138]. The way these receptors tend to dimerize is substantial, as molecules inhibiting dimerization may be plausibly used in anticancer therapy. Intriguingly, the possibility of EGFR^vIII^*cis-*autophosphorylation is very rarely discussed in the literature [135, 141]. Cross-activation of EGFR^WT^ kinases is well recognized as the mechanism crucial for their activation, clearly explaining why receptor dimerization is actually required. Nevertheless, *a priori* rejection of the hypothesis stating that some part of EGFR^vIII^*cis*-autophosphorylates may be too hasty.

EGFR^vIII^ is also suggested to dimerize with monomers of other inactive receptors (hepatocyte growth factor receptor, HGFR; platelet-derived growth factor receptor, PDGFR) or to have an indirect impact on their function (Figure 2(c)) [142, 143]. Regulation of other receptors may potentially constitute an additional mechanism for EGFR^vIII^-mediated activation of signal transduction pathways, especially in the absence of ligands. This hypothesis was more profoundly tested by Greenall et al., who demonstrated that EGFR^vIII^ is able to activate HGFR via focal adhesion kinase (FAK); however, it was not clearly defined how such activation takes place on a FAK protein platform [139].

All doubts concerning the mechanism of EGFR^vIII^ encourage researchers to search for mechanisms of oncogenic action of this protein that are independent of its membrane receptor activities. One of the most interesting analyses is focused on the nuclear role of EGFR^vIII^, as this function is suggested to be very relevant [144, 145]. Therefore, EGFR^vIII^ interaction with oncostatin M receptor (OSMR) can be considered interesting, as it may be possible to design molecules inhibiting such interaction for therapeutic purposes [146]. In general, results of research conducted so far indicate that the majority of EGFR^vIII^ activity is exhibited outside the nucleus, while its low kinase activity may be compensated by uniquely high stability [17, 121, 123–125, 147–149].

## 4. Biological Role of EGFR^vIII^

Despite the fact that cells with high expression of mutated receptor are unable to bind EGFR ligands, these cells are still characterized by increased invasiveness and enhanced proliferation rate when compared to cells with low EGFR^vIII^ expression or EGFR^vIII^-negative ones [147]. Therefore, it is not only the mechanism of EGFR^vIII^ action, but also biological changes triggered within EGFR^vIII^-positive cells, as well as the role such cells play in tumor as a whole, that are important when considering the impact of this mutated receptor. This aspect can be especially important, since EGFR^vIII^ expression is not observed in all cells comprising the tumor [33]. Our data suggested that EGFR^vIII^ acts as a classical oncogene, stimulating proliferation and inhibiting apoptosis of glioblastoma cells [147], while other studies indicated much more complicated influence of this oncogene. Considering the impact of EGFR^vIII^ on cells, both autocrine and paracrine effects were investigated. As an example, EGFR^vIII^-positive cells may secrete leukemia inhibitory factor (LIF) and IL-6 that activate IL-6R/gp130 receptors present on the surface of EGFR^WT^-positive cells, promoting their proliferation. Moreover, by activation of NF-κB pathway and stimulation of survivin expression, IL-6 may make cells more resistant to apoptosis [150, 151].

EGFR^vIII^ amplicons are present only in part of glioblastoma cells derived from patients and in stable DK-MG cell line (intratumoral heterogeneity). Moreover, only part of these amplicons is active (not epigenetically silenced) and enables expression of the mutated gene [31]. EGFR^vIII^ expression alone is epigenetically controlled, as it was demonstrated that inhibition of histone deacetylation leads to decrease in expression of this oncogenic receptor. It may be explained by the fact that there is a relatively low EGFR^vIII^ expression in tumor parts where high amplification level of this mutated gene is detected [31, 152].

Some researchers suggest that EGFR^vIII^ expression may be present on the surface of brain cancer stem-like cells (bCSCs) that share some similarity with normal neural stem cells (NSCs) [153, 154]. The former cells are characterized by self-renewal potential as well as expression of markers characteristic for stem cells [155–157]. EGFR^vIII^ is coexpressed with marker characteristic for nondifferentiated cells (CD133 and SOX2) [158, 159], and it is even indicated that this oncogenic receptor may be used to define CSC populations [158]. One can speculate that low kinase activity together with high stability of EGFR^vIII^ is enough to inhibit cell differentiation. Interestingly, it can be also assumed that EGFR^vIII^ epigenetically reprograms cells, depriving them of differentiation potential and, hence, following such process, this mutated receptor may be no longer needed. Brain CSCs are involved in initiation and progression stages of GB, mostly due to their impact on angiogenesis and treatment response [160, 161]. Moreover, presence of bCSCs may hinder long-term maintenance of therapeutic effect, as currently used compounds do not affect these cells, mostly due to very efficient DNA damage repair mechanisms [160, 161]. However, it was demonstrated that usage of bispecific antibodies directed against EGFR^vIII^ and CD133 (CSCs marker) has a cytotoxic effect on bCSCs and impairs their self-renewal abilities [158]. Some researchers suggest that *in vivo* CSCs, but not other cancer cells, are mostly responsible for the process of tumor formation in SCID mice as well as for the propagation of intratumoral heterogeneity [162]. Our results clearly demonstrated SOX2 expression in high percentage of GB cells that, in our opinion, undermines the presence of only a minor stem cell population in glioblastoma tumors [163].

## 5. Intratumoral Heterogeneity of Glioblastoma in terms of EGFR^vIII^ Expression

**Figure 3:**
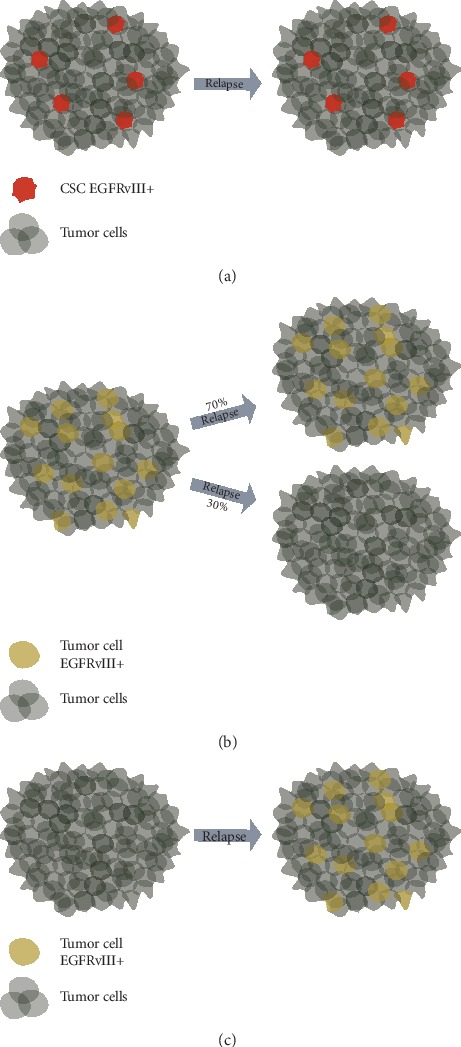
Hypotheses concerning the presence and role of EGFR^vIII^-positive cells in tumors, on the example of glioblastoma. (a) One of the hypotheses states that EGFR^vIII^ is expressed on the surface of cancer stem cells (CSCs). In such a case, EGFR^vIII^-positive CSCs should be also detected in recurrent GB tumors [164]. Nevertheless, failure to detect such cells may be due to the exposure of primary tumor to therapeutic compounds. (b) Another hypothesis states that EGFR^vIII^-positive cells are only crucial during the early stages of carcinogenesis. It is supported by reports demonstrating loss of expression of this mutated oncogene in approx. 30% of patients with EGFR^vIII^-positive primary tumors [119, 165]. (c) Cells expressing EGFR^vIII^ are also reported in recurrent tumors when primary GB was EGFR^vIII^-negative [119, 165].

The fact that EGFR^vIII^ is not present in all GB cells in tumor mass may complicate the perception of this receptor as a perfect therapeutic target. However, if cells expressing EGFR^vIII^ are cancer stem cells [164] or EGFR^vIII^-negative cells are somehow dependent on EGFR^vIII^-positive ones, then discussed targeted therapy may turn out to be effective (Figure 3(a)). Our research indicates that EGFR^vIII^-negative cells may be indeed dependent on EGFR^vIII^-positive population. It is supported by the fact that we were unable to establish a subline of DK-MG cell line completely deprived of cell expressing this mutated oncogene, as at least small percentage of EGFR^vIII^-positive cells was necessary in order to maintain survival and proliferation [33, 147]. On the other hand, at least in 30% of cases, EGFR^vIII^ expression is spontaneously lost in recurrent GB tumors, even when the treatment was not directed against the mutated receptor (Figure 3(b)) [119, 165]. Remarkably, there were also some cases in which EGFR^vIII^ expression was detected only in recurrent GB tumors (Figure 3(c)) [119, 165]. Such observations are of utmost importance, as these enable to evaluate the relevance of EGFR^vIII^ and indirectly cells expressing this mutated receptor, as therapeutic targets. If EGFR^vIII^ is lost (not detected) in recurrent tumors due to the fact that it is present only in a small part of cells and EGFR^vIII^-negative cells are independent of the activity of this oncogenic variant, it undermines the validity of EGFR^vIII^-targeting therapies, for example, those based on CAR-T technology [166]. It may be associated with the fact that the expression of some oncogenes, including EGFR^vIII^, is crucial at earlier stages of neoplastic transformation, but not further during advanced cancer progression. Opinions on the role of EGFR^vIII^ as well as EGFR^vIII^-positive cells are extremely different, as this oncogene is suggested either to play an insignificant role at the later stages of carcinogenesis, or, on the contrary, to be a marker of GB stem cells (Figures 3(a)–3(c)). Our analyses do not confirm the hypothesis stating that EGFR^vIII^ is irrelevant in fully differentiated GB cells, as DK-MG cells deprived of this oncogene expression lose their proliferation abilities are more prone to apoptosis and unable to give rise to tumors in SCID mice models [147].

Recently, a lot of attention is focused on the ability to transfer extrachromosomal vesicles containing various structures (including DNA amplicons) between cells. Obviously, extrusion of amplicons or decrease in their number during mitoses may lead to generation of cells without amplicons [120]. Simultaneously, amplicons may be transferred to cells initially lacking such structures. Derivation of amplicon-deprived cells from cells with amplicons, as well as “infection” of cells lacking amplicons with these elements of extrachromosomal DNA, is in favor of hypothesis stating that EGFR^vIII^-positive cells may, in a certain sense, play a role of precursor cells. It clearly emphasizes the biological role of EGFR^vIII^ not at the protein, but DNA level, and it may partially explain why the expression of this mutated protein is in particular cases very low, almost at the detection level of protein analysis methods such as western blot. However, it should not be confused with the role played by so-called cancer stem cells.

The fact that intratumoral heterogeneity may constitute one of the mechanisms responsible for resistance of cancer cells to targeted therapies (including TKIs) was first demonstrated by Nathanson et al. and further confirmed by other research teams. Such a specific adaptation via changing cell phenotypes is mainly focused on achievement of an optimal balance for the unaltered proliferation of the overall population and is mostly due to dynamic regulation of extrachromosomal DNA encoding mutated EGFR [167–169].

## 6. Targeted Therapies Based on Tyrosine Kinase Inhibitors (TKI), Directed against EGFR^WT^ and EGFR^vIII^

A wide variety of factors contribute to the fact that glioblastoma is one of the most difficult tumors from a clinical perspective and that effective therapies for patients diagnosed with this tumor type are still lacking. So far, many therapeutic approaches were developed to treat patients with EGFR^vIII^-positive glioblastoma (Table 1). Generally, average survival rate of GB patients does not exceed 12–14 months from the moment of diagnosis and there has been actually no improvement for many years [170]. Ideal drug directed against GB cells should be well tolerated by the patients, able to cross the blood-brain barrier, and specifically induce tumor cell death. Classical therapeutic regimen in case of GB consists of surgical resection with adjuvant radio- and chemotherapy with alkylating agent temozolomide [171]. Current clinical and preclinical trials concerning anti-EGFR/EGFR^vIII^ therapies include small molecule tyrosine kinase inhibitors, antibodies, vaccines, as well as therapies based on RNA interference. As silencing of a single gene in a particular signaling pathway may not be sufficient to provide a therapeutic effect in GB patients, there is a need for a complex approach, focusing on several signal transduction pathways [172].

There have been several attempts to experimentally apply EGFR tyrosine kinase inhibitors, also inhibiting EGFR^vIII^, in glioblastoma therapy, as significant differences between kinase domains of mutated and wild-type receptor have not been described so far. A broad spectrum of anti-EGFR TKIs was developed, with the first-generation inhibitors (gefitinib, lapatinib, and erlotinib) binding reversibly and the second-generation inhibitors (afatinib and dacomitinib) binding covalently to the receptor [173–175]. Inhibitors of the third generation (rociletinib and osimertinib) covalently bind to ATP-binding site in cells with T790M EGFR mutation, conferring resistance to inhibitors of previous generations [176]. Second-phase clinical trial studies demonstrated that gefitinib, lapatinib, and erlotinib administered to patients with primary or recurrent GB tumors resulted in only marginal therapeutic response, when administered either in monotherapy or in combination [72, 77, 177]. Although osimertinib may be recognized as especially important in terms of EGFR^vIII^, as it was suggested to be efficiently delivered to cancer cells in brain [176], the activity of this compound against EGFR^vIII^-positive cells was lower when compared to afatinib. Since the kinase domain of this splice variant is structurally close to EGFR wild type, this was not unexpected [176].

In case of TKI-based therapy, alterations downstream to EGFR^vIII^, including PTEN mutations, should be taken into account. Despite the fact that inactivating mutations of PTEN have an impact on only one of EGFR-regulated pathways (AKT), it was demonstrated that such mutation is able to hinder the impact of erlotinib on GB cells [178]. Considering this aspect, immunotherapies may possibly outperform small molecule-based approaches. Despite the wide availability of TKIs clinically approved in oncological treatment, none of these inhibitors is used as a standard approach in GB treatment [64, 72, 77, 179]. As EGFR^vIII^ is a key oncogene with kinase activity-dependent function, it seems reasonable to consider whether the efficacy of TKI-based therapies should not be greater, especially since it has been postulated that the blood-brain barrier in advanced GB is disrupted and thus should not enable for crossing of small molecules [180]. It is well established that EGFR-targeting TKIs improve the progression-free survival of patients with EGFR-mutated non-small-cell lung cancers (NSCLCs) [181, 182]. Therefore, the verification whether glioblastoma patients with high frequency of EGFR mutations respond to TKIs is completely justified, even despite different EGFR mutational spectrum. This becomes even more important since Orellana et al. showed that ectodomain EGFR mutations including those leading to EGFR^vIII^ may sensitize tumor cells to tyrosine kinase inhibitors [183]. Reports from *in vitro* studies conducted on EGFR^vIII^-expressing cell lines tend to be contradictory. Some results indicate EGFR^vIII^ sensitivity, while the others demonstrate that EGFR^vIII^, in contrast to EGFR^WT^, appears to be relatively resistant to EGFR-TKIs [87]. By now, several TKI-involving clinical trials on glioblastoma were completed or terminated, however still without any significant patients' benefits [65, 72, 77, 88, 178, 184–188]. Moreover, it was demonstrated that although cetuximab binds to EGFR^vIII^ and decreases expression and leads to overall downregulation of this mutated receptor, it does not inhibit the proliferation of EGFR^vIII^-expressing GB cells and is not effective in GB clinical trials [189–191]. Therefore, it seems that so far neither EGFR-TKIs nor monoclonal antibodies such as cetuximab are effective therapeutic options in glioblastoma patients, irrespective of EGFR^vIII^ occurrence in tumor [177, 188]. Thus, it is difficult to speculate whether EGFR^vIII^ affects EGFR-targeted treatment, as no treatment approach was truly effective in patients, in spite of quite encouraging results from *in vitro* studies. As EGFR^vIII^ presence in other tumor types is highly debatable, there were no clinical trials to investigate the issue of EGFR^vIII^-modulated TKI treatment response in tumors other than glioblastoma. Hence, conclusions from studies on EGFR-TKIs/immunotherapies can only be drawn concerning this particular tumor. Finally, as Orellana et al. recently suggested the high probability that mutated ectodomain of EGFR^vIII^ induces structural changes in the intracellular kinase domain [183], further research focused on detailed understanding of molecular aspects of EGFR^vIII^ should be expected. On the other hand, considering current standard therapeutic GB regimen, EGFR^vIII^ is associated with prolonged survival of GB patients treated with surgery and radio/chemotherapy [192]. It was clearly shown that cases of MGMT-methylated GB with endogenous EGFR^vIII^ expression are significantly more sensitive to temozolomide, than their isogenic EGFR^vIII^ -negative counterparts [193].

## 7. Immunotherapy in EGFR^vIII^-Positive Tumors

Apart from TKIs, antibodies constitute the most extensively analyzed group of EGFR-targeting compounds; however, their evident efficacy in GB has not been demonstrated so far. High molecular weight may be one of the factors limiting their applicability in treatment of this tumor type [194], but the integrity of blood-brain barrier may be compromised in case of tumors with high level of malignancy [180]. Cetuximab is a chimeric monoclonal IgG1 antibody directed against extracellular domain of EGFR that in clinical studies was demonstrated to exert anticancer effect and increase tumor cell sensitivity to radiotherapy in GB [92]. This molecule was approved by the Food and Drug Administration for the treatment of patients with head and neck cancer and advanced colon cancer. Interestingly, it may be used in case of increased expression of both EGFR^WT^ and EGFR^vIII^ [195, 196], as it was demonstrated that cetuximab may bind to domain III (L2) of EGFR^vIII^ and reduce autophosphorylation of this mutated receptor [197]. Preclinical analyses indicate that following EGFR^vIII^ binding cetuximab induces receptor internalization, resulting in 50% reduction of its active form [197]. Nevertheless, there is a lack of clinical studies evaluating the impact of cetuximab monotherapy on patients with primary glioblastoma [198, 199]. When tested *in vitro* on GB cell lines with EGFR overexpression or using *in vivo* GB models, cetuximab leads to decrease in proliferation rate and enhancement of apoptosis. Additionally, in the latter model, this antibody is able to significantly inhibit tumor growth and increase median survival rate [93]. During analyses conducted using stable cell lines as well as neurospheres, magnetic iron oxide particles (IONPs) were used to increase therapeutic availability of cetuximab and resulted in more effective binding of antibody to GB cells when compared to cetuximab alone, as evaluated by the inhibition of EGFR signaling pathway and increased receptor internalization [200]. There is also an ongoing research on the use of other antibodies in GB therapy, for example, panitumumab (humanized monoclonal IgG2 antibody) or nimotuzumab (humanized monoclonal IgG1 antibody), that are functionally similar to cetuximab [109, 201]. These antibodies also bind to L2 domain, preventing ligand binding and receptor dimerization [202]. Randomized phase III clinical trials demonstrated that nimotuzumab administration in adult GB patients increases overall survival when compared to standard treatment [109].

In order to achieve higher therapeutic response, it is also possible to conjugate antibodies with other drugs (antibody drug conjugates, ADC). So far, ABT-414 and AMG-595 were developed [114, 203] and the former conjugate was demonstrated to selectively induce apoptosis in cells with EGFR^WT^ overexpression or EGFR^vIII^ expression both *in vitro* and *in vivo* using xenograft models. ABT-414 conjugate consists of ABT-806 monoclonal antibody directed against EGFR and inhibitor of microtubule polymerization—monomethyl auristatin F. Despite the fact that ABT-806 was initially developed to specifically interact with EGFR^vIII^, it also binds to wild-type receptor, however, to a lesser extent [204]. Using xenograft GB models, it was demonstrated that combination of ABT-414 with standard chemo- and radiotherapy resulted in a significant decrease in cell proliferation and overall decrease in tumor growth [114]. Currently, there are ongoing phase I/II clinical trials aimed at evaluating the efficacy of ABT-414 administration in patients with newly diagnosed (NCT02573324) or recurring GB (NCT02343406). Analyses on an orthotopic mouse GB model showed that ind-111-labeled ABT-806 antibody can specifically recognize cancer cells [205].

Nowadays, one of the most promising immunotherapy-based approaches in GB treatment is the usage of autologous T lymphocytes with chimeric antigen receptor—CAR-T cells. These are T lymphocytes that have been modified *ex vivo* and able to recognize their molecular target irrespective of antigen presentation by the molecules of major histocompatibility complex [52, 206]. Structure of CAR-T cells makes them able to exhibit both activity of antibodies and toxicity of T lymphocytes [207]. CAR-T is a technology of interest in research on many cancer types; however, the prerequisite for its efficacy and lack of side effects is antigen expression specifically on cancer cells [208]. As EGFR^vIII^ meets this requirement, it is possible to develop CAR-T recognizing mutated form of the receptor by antigen-specific, humanized single chain of variable fragment of antibody, conjugated with transmembrane and intracellular domains of T lymphocytes and NK cells. Similarly to modified T lymphocytes, NK cells with introduced CAR are able to exhibit cytotoxic activity *in vitro* [209]. CAR-T cells directed against EGFR^vIII^ were able to effectively infiltrate tumor cells in brain in *in vivo* model [53]. Notably, EGFR^vIII^-targeting CAR-T therapies have currently reached phase I of clinical trials to treat GB patients (NCT03283631, NCT02209376). On the other hand, administration of CAR-T therapy in phase I and I/II clinical trials directed against antigens present on both normal and cancer cells (ErbB2, CD19) led to severe side effects and even patients death [210, 211]. It is still not clear whether antibodies designed to recognize EGFR^vIII^ also detect wild-type EGFR [43, 205, 212, 213], but if so, it may lead to some serious side effects following administration of various immunotherapies, including CAR-T approach. Moreover, as glioblastoma tumor is highly heterogeneous, not all GB cells may respond to CAR-T therapy directed against EGFR^vIII^.

Apart from CAR-T technology, there is also an ongoing research on application of another immunotherapy-based approach in GB treatment in a form of bispecific antibodies activating T lymphocytes—bispecific T-cell engagers (BiTEs). BiTEs are recombined immunoglobulins composed of a single chain of variable fragments of two antibodies: one directed against antigen expressed on the surface of T lymphocytes and the second one against the antigen present on target cells [214, 215]. Heavy and light chains of variable antibody fragments are connected with a short, elastic linker, rich in glycine and serine residues. Extracellular EGFR^vIII^ domain is small; hence, it may be efficiently bound by BiTEs [216], and specificity and cytotoxic activity of these molecules against this mutated receptor were demonstrated using *in vitro* and *in vivo* models. Properly designed bispecific antibodies are characterized with very high specificity, resulting in a minimal risk of induction of cross reactions in normal cells [55]. Using BiTEs, it was demonstrated that stimulated regulatory T lymphocytes secrete elevated levels of granzymes and perforins and that their activity is directed against EGFR^vIII^-positive cells [217, 218]. Currently, there is an ongoing phase I clinical trial on administration of AMG 596, drug containing BiTEs directed against EGFR^VIII^ and CD3 surface protein in GB patients (NCT03296696).

It is worth to emphasize that results of our analyses, supported by the data gathered by other research teams, indicate that additional mutations within EGFR^vIII^ that may have an impact on efficacy of antibodies or small molecules directed against EGFR^vIII^-characteristic protein fragments are rarely occurring, but if so, these are distant from EGFR^vIII^-specific parts [219, 220].

Vaccines constitute another therapeutic approach taking advantage of patient's immunological system to destroy EGFR^vIII^-positive cells. So far, only one peptide vaccine, rindopepimut, has been developed to induce humoral response leading to elimination of GB cells expressing mutated EGF receptor [221]. Rindopepimut (CDX-110) is based on 13-amino acid EGFR^vIII^-specific sequence conjugated with keyhole limpet hemocyanin (KLH; hemocyanin neoantigen) adjuvant [222]. *In vivo* preclinical analyses demonstrated that tumor volume significantly decreased in 70% of animals with subcutaneously injected cancer cells following CDX-110 administration, when compared to the control group. It was suggested that antibodies reactive against EGFR^vIII^-KLH are involved in triggering of antibody-dependent cell cytotoxicity (ADCC), regardless of antigen-specific T lymphocytes activity [223]. Median survival rate of GB patients treated with CDX-110 after surgical resection and chemotherapy was prolonged to 24 months, as demonstrated in 3 independent phase II clinical trials (ACTIVATE, ACT II, and ACT III). Moreover, EGFR^vIII^-expressing cells were not detected in 67% of patients receiving CDX-110 treatment for at least 3 months [222]. Nevertheless, phase III clinical trial (ACT IV), comparing the efficacy of temozolomide alone and temozolomide in combination with CDX-110 in GB, was terminated before the scheduled date, as despite premises from the previous stages of clinical trials, it failed to indicate the significant increase in patient survival (median survival for CDX-110-treated patients was 20.1 months, while in control group 20 months). Still, the researchers emphasized the relevance of research focused on determination of the type of immunological response induced by CDX-110 and highlighted the problem of the selection of the appropriate molecular target for immunotherapy approaches [58].

## 8. Anti-EGFR^vIII^ Therapy Based on RNA Interference

Concerning the regulation of EGFR^vIII^ expression, emphasis should be also put on noncoding RNAs, especially microRNAs (miRNAs). Aberrant expression of miRNAs has been implicated in various tumor types, including glioblastoma, and demonstrated to impact cancer cell proliferation, EGFR downstream signaling, as well as efficacy of several anti-EGFR-targeting therapeutic approaches. Unfortunately, the majority of data were focused on wild-type receptor and we can only speculate that similar mechanisms apply to EGFR^vIII^. Decrease in miR-137 level in glioblastoma tissue samples was found to be associated with poor prognosis and, consequently, overexpression of this miRNA in GB models resulted in elevated apoptosis and inhibition of tumor cell growth. It was suggested that miR-137 may act by decreasing translation of EGFR protein, hence decreasing proproliferative activity of this receptor in tumor cells [224]. Similarly, miR-615, miR-1231, or miR-133, also downregulated in glioblastoma, were found to inhibit EGFR levels [225–227]. On the other hand, upregulation of miR-21, often found in glioblastoma patients, promotes EGFR activity and supports tumor growth [228]. Yin et al. showed that miR-34a was often deleted in glioblastoma showing EGFR amplification; however, they did not evaluate the expression of EGFR^vIII^ within analyzed samples. Nevertheless, considering the typical percentage of EGFR^vIII^-positive GB cases with EGFR amplification, it is very likely that miR-34a deletion coexists with EGFR^vIII^. Notably, Yin et al. indicated shorter mean survival rate of patients diagnosed with GB with EGFR amplification and miR-34a deletion compared to patients with only one of these alterations [229]. Moreover, EGFR^vIII^-mediated downstream signaling was found to be associated with inhibition of miR-9 expression, further promoting tumorigenicity in FOXP1-dependent manner [230]. Intriguingly, lncRNA EGFR-AS1 was found to act via miR-133b in regulation of glioblastoma cell migration, invasion, and apoptosis and knockout of this noncoding RNA negatively influenced tumor growth [231].

Besides protein-based therapeutic approaches, research focuses on targeting EGFR^vIII^ at the mRNA level. RNA interference-based therapy relies on usage of ribozymes, antisense oligonucleotides, or siRNA molecules complementary to regions that silencing is beneficial from a clinical point of view [61]. Taking advantage of this technology enables to inhibit activity of EGFR signaling pathways, with relatively low toxicity and maintained high specificity against EGFR^vIII^ [63, 232, 233]. As promising results were obtained in preclinical analyses with antisense oligonucleotides for the treatment of non-small-cell lung carcinoma and prostate cancer [234, 235], possibility to silence EGFR and EGFR^vIII^ gained more attention. Sequence of mRNA nucleotides in junction site between introns 1 and 7 in EGFR^vIII^ is highly specific and absent in any other human genes. Nevertheless, the majority of current literature data concerning siRNA is focused on EGFR in general, without distinguishing normal receptor from mutated one. Constructs with proper antisense RNA sequence were demonstrated to silence expression of mRNA encoding EGFR^WT^, both *in vitro* on GB cells with EGFR^WT^ expression and *in vivo* on rat GB model. In the former model, significant decrease in level of EGFR mRNA and protein, decrease in proliferation rate, and induction of apoptosis were observed in cells with the expression of introduced construct, while in the latter model all rats with introduced antisense RNA were characterized with prolonged survival rate, when compared to animals with empty construct [236]. Comparison of the construct with antisense RNA complementary to 3′ end and to the whole EGFR^WT^ mRNA encoding region demonstrated that inhibition is more effective in the first case, possibly as delivery of shorter construct may be much easier and efficient [236, 237]. First reports indicate that siRNA complementary to exon 1 and 8 junction site is able to inhibit EGFR^vIII^ expression in human glioma cells, leading to decrease in AKT phosphorylation and inhibition of cell cycle in G2/M [238].

Gene therapy using ribozymes is based on the ability of antisense RNA to catalytically digest mRNA substrate within the specific nucleotide sequence [239]. Low-molecular hairpin-type ribozymes were able to specifically inhibit EGFR expression, as well as proliferation and clonogenicity of GB cells *in vitro* [60, 239]. In terms of gene-editing approaches, it is worth to mention that CRISPR-based technologies have only little chance of being successfully applied in case of EGFR^vIII^, as deletions within EGFR leading to the formation of this oncogenic variant are quite extensive and tend to differ between patients [240]. It is worth to mention that RNA interference can be achieved by miRNA upregulation. Moreover, one of the miRNAs, miRNA-34a, was demonstrated to enhance the antiproliferative effect of erlotinib [241].

## 9. In Vitro Models for EGFR^vIII^ Analyses

One of the additional and still unresolved problems regarding development of an effective anti-EGFR^vIII^ therapy is lack of the appropriate *ex vivo*/*in vitro* models reflecting heterogeneity of GB cell genotype and phenotype. Results obtained under *in vitro* conditions often tend to differ significantly from those obtained in clinical trials, as exemplified by results presented above. In primary GB cultures, EGFR^vIII^ expression is quite stable in neurospheres, while in adherent cultures it tends to be lost as soon as after several passages. Analyses of SOX2 expression (marker of neural stem cells and factor crucial to maintain proliferation of GB cells) indicate that neurospheres and adherent cells differ in the state of differentiation—adherent cells gradually lose SOX2, while in spheroids expression of this marker remains at relatively constant level [242]. It is worth to emphasise that the majority of GB cells are SOX2-positive, as it is in contrary to the assumptions that cancer stem cells constitute only so-called side population or, it is possible that SOX2 is a marker not characteristic solely for stem cells [243, 244]. Apart from SOX2, glioblastoma cells also express GFAP, which can be considered quite surprising as GFAP for many years has been considered a marker of mature astrocytes. Nevertheless, GFAP-positive neural stem cells have been described in the literature [245] and these, similar to GB cells, were demonstrated to coexpress many other markers [163, 245].

Despite the fact that spheroid cultures maintain original phenotype of GB cells for a longer period (there is no stable GFAP^+^/SOX2^+^ adherent cell line), this approach is associated with various methodological difficulties. First of all, certain assays on 3D structure may be difficult to be performed. Moreover, cells maintained in medium containing serum are more resistant to the exposure to cytotoxic molecules than neurospheres cultures in serum-free media. Finally, not all primary GB cells are able to form spheroid structures [246]. Basically, *in vitro* culturing should promote survival and proliferation of cancer cells; however, it may lead to spontaneous senescence, mitotic catastrophe, or apoptosis. The occurrence of *in vitro* senescence described to play both pro- and antineoplastic role *in vivo* in primary GB cultures can be plausibly associated with failure in their stabilization [247]. Stable glioblastoma line may not only fail to reflect the heterogeneous nature of tumor cells observed *in vivo*, but also lack extrachromosomal amplicons encoding EGFR^vIII^ [21, 150, 167]. However, the limited amount of tumor material derived from patients and its low stability force scientists to conduct research on commercially available stable cell lines with exogenously introduced EGFR^vIII^-encoding gene [153, 248]. Analyses on such models may be unreliable, as introduction of EGFR^vIII^ cDNA via cell engineering methods may give biased results regarding such aspects as clonality (different results obtained depending on the analyzed clone) or neglect the dynamic regulation of amplicons released from EVs. Additionally, exogenously introduced EGFR^vIII^ may not have an impact on the biology of already fully defined cancer cells, such as U87-MG cell line [147]. Therefore, biological differences observed between U87-MG clones may be easily confused and taken as the effect of EGFR^vIII^ action. Hence, there is an ongoing search for the most appropriate model, reflecting nature of GB cells as precisely as possible.

## 10. Summary

Table 2 presents most important issues addressed in the article (except therapies in Table 1). EGFR^vIII^ protein may be considered a suitable target in 28–30% of GB cases, as it is selectively expressed on cancer cells and structurally differs from wild-type receptor. Nevertheless, opinions on the role of EGFR^vIII^ in GB biology are contradictory. This mutated receptor seems to play a key role in tumor cells, enhancing their proliferation, inhibiting apoptosis, or being considered a marker of CSCs. On the other hand, it is suggested that EGFR^vIII^ is unnecessary for GB cells, especially at advanced stages of tumorigenesis, that may be considered a drawback in terms of therapeutic approaches directed against this mutated receptor. Despite many years of extensive research, EGFR^vIII^-specific inhibitors have not been developed yet. There are also many controversies regarding antibodies designed to specifically detect this oncogenic variant, which in turn may be negatively correlated with the efficacy of CAR-T and other immunotherapy-based approaches. Many factors hinder glioblastoma treatment, including heterogeneity of EGFR^WT^/EGFR^vIII^ expression, the impact of receptor signaling on various cellular processes, mechanisms of cells resistance to treatment, or the presence of cancer stem cell populations. Undoubtedly, anti-EGFR^vIII^ therapies constitute the important area of research, but the structure, mechanism of action, and the biological role of EGFR^vIII^ need to be determined for their proper development. In particular, it is crucial to resolve whether EGFR^vIII^-negative glioblastoma cells are dependent on EGFR^vIII^-positive population or not.

## Figures and Tables

**Table 1:** Agents acting specifically on EGFR^vIII^ or on both EGFR^vIII^ and EGFR^WT^, based on the analysis of different cancer types.

		Specificity	Examined cancers	Activity	Stage of research	References
*Agents acting only on EGFR* ^*vIII*^						
Immunotherapy						
ADC	AMG-595	EGFR^vIII^	Glioblastoma	Potentially active	Phase I	[51]
CARs	CAR-T	e.g., EGFR^vIII^	Glioblastoma	Potentially active	Phase I	[52, 53]
			Lung cancer	Potentially active	Preclinical	[54]
BiTE	bscEGFR^vIII^ × CD3	e.g., EGFR^vIII^	Glioma	Potentially active	Phase I	[55, 56, 57]
Vaccine	Rindopepimut	EGFR^vIII^	Glioblastoma	Inactive	Phase III	[58]

RNA interference						
Ribozymes		e.g., EGFR^vIII^	Breast cancer	Potentially active	Preclinical	[59]
			Glioblastoma	Potentially active		[60]
Antisense oligonucleotides		e.g., EGFR^vIII^	Glioblastoma	Potentially active	Preclinical	[61, 62]
siRNA		e.g., EGFR^vIII^	Glioblastoma	Potentially active	Preclinical	[61, 63]

*Agents acting on EGFR* ^*vIII*^ *and EGFR* ^*WT*^						
Tyrosine kinase inhibitors						
First generation	Gefitinib	EGFR/HER1	High-grade gliomas	Limited activity	Phase II	[64, 65]
			Non-small-cell lung cancer	Active	Clinical use	[66, 67]
			Salivary gland cancer	Potentially active	Phase II	[68]
			Breast cancer	Potentially active	Phase II	[69]
			Ovarian, peritoneal, or fallopian tube cancer	Potentially active	Phase I/II	[70]
			Liver cancer	Potentially active	Phase II	[71]
	Lapatinib	EGFR/HER1/HER2	Glioblastoma	Inactive	Phase I/II	[72, 73]
			Breast cancer	Active	Clinical use	[74]
			Gastric cancer	Limited activity	Phase II	[75]
			Colorectal cancer	Potentially active	Phase II	[76]
	Erlotinib	EGFR/HER1	Gliomas	Limited activity	Phase II	[77, 78]
			Vulvar cancer	Potentially active	Phase II	[79]
			Non–small-cell lung cancer	Active	Clinical use	[80, 81]
			Pancreatic cancer	Active	Clinical use	[82]
			Head and neck cancer	Limited activity	Phase II	[83, 84]
Second generation	Afatinib	EGFR/HER1/HER2/HER4	Non-small-cell lung cancer	Active	Clinical use	[85]
			Squamous cell carcinoma of the lung	Active	Clinical use	[85]
			Head and neck cancer	Potentially active	Phase III	[86]
			Glioblastoma	Limited activity	Phase I/II	[87, 88]
			Breast cancer	Potentially active	Phase II	[89]
			Colorectal cancer	Potentially active	Phase II	[90]

Immunotherapy						
Antibodies	Cetuximab	EGFR/HER1/HER2	Head and neck cancer	Active	Clinical use	[91]
			Glioblastoma	Potentially active	Phase II	[92, 93, 94]
			Colorectal cancer	Active	Clinical use	[95]
			Esophageal and gastric cancer	Limited activity	Phase II	[96]
			Non-small-cell lung cancer	Potentially active	Phase II	[97]
			Breast cancer	Limited activity	Phase II	[98]
			Prostate cancer	Inactive	Phase II	[99]
			Cervical cancer	Inactive	Phase II	[100]
	Panitumumab	EGFR/HER1	Colorectal cancer	Active	Clinical use	[101, 102]
			Biliary tract cancer	Potentially active	Phase II	[103]
			Head and neck cancer	Inactive	Phase II	[104, 105]
			Glioblastoma	Potentially active	Phase II	[106, 107]
			Breast cancer	Potentially active	Phase II	[108]
	Nimotuzumab	EGFR/HER1	Glioblastoma	Orphan status in Europe and USA	Clinical use	[109, 110]
			Head and neck cancer	Active	Phase II	[111, 112]
			Pancreatic cancer	Orphan status in Europe	Clinical use	[110, 113]

ADC	ABT-414	EGFR/EGFR^vIII^	Glioblastoma	Limited activity	Phase I	[114, 115]
			Breast cancer	Limited activity	Phase I/II	[116]

**Table 2:** Issues addressed in the article (except therapies in Table 1).

EGFR^vIII^ issue/process	Mechanism/way to address	Selected references
EGFR^vIII^ presence in tumors/cancers	GB in about 40%, rarely in HNCSCC, lung prostate, colorectal cancer, breast cancer	[27, 33–41, 43]
EGFR^vIII^ mechanism of mutation	Deletion of EGFR exons 2–7	[26–29, 43]
EGFR^vIII^ mechanism of action	Several models: (1) Heterodimerization with EGFR^WT^ (2) Homodimerization (3) EGFR^vIII^ and MET cooperation, FAK involved (4) OSMR mechanism Resistant to degradation important for all models	[16, 17, 121–126, 142, 143]
EGFR^vIII^ biological role	Extreme opinions: from lack of important role at advanced cancer (tumor) stages, to role in self-renewal, survival, and proliferation of cancer stem cells	[33, 147, 150, 152–158, 164, 165]
EGFR^vIII^ cell culture models	3D primary cell cancer cell models, DK-MG model, genetically modified cancer cell lines	[21, 150, 153, 167, 242–244, 246–248]
